# An algorithm for the characterization of influenza A viruses from various host species and environments

**DOI:** 10.1111/irv.13258

**Published:** 2024-02-22

**Authors:** Laura A. Pulscher, Richard J. Webby, Gregory C. Gray

**Affiliations:** ^1^ Department of Medicine (Infectious Diseases) University of Texas Medical Branch Galveston Texas USA; ^2^ Department of Infectious Disease St. Jude Children's Research Hospital Memphis Tennessee USA; ^3^ Department of Microbiology and Immunology University of Texas Medical Branch Galveston Texas USA; ^4^ Institute for Human Infections and Immunity University of Texas Medical Branch Galveston Texas USA; ^5^ Center for Tropical Diseases University of Texas Medical Branch Galveston Texas USA

**Keywords:** influenza a virus, laboratory diagnosis, molecular diagnostic testing, *Orthomyxoviridae*

## Abstract

Due to the extensive host range of influenza A viruses, it is difficult to determine the best diagnostic algorithm to efficiently screen samples from a variety of host species for influenza A viruses. While there are some influenza diagnostic algorithms that are specific to host species, to our knowledge, no single algorithm exists for the characterization of influenza A viruses across multiple host species. In this paper, we propose an algorithm that can serve as a guide for screening human, animal, and environmental samples for influenza A viruses of high human and animal health importance.

## 1 INTRODUCTION

Influenza A viruses (*Alphainfluenzavirus influenzae*) in the family *Orthomyxoviridae* are notably one of the most important causes of respiratory disease among both humans and animals. These negative‐sense, single‐stranded RNA viruses have a segmented genome and are subtyped based on their hemagglutinin (HA) and neuraminidase (NA) glycoproteins, of which there are currently 16 HA and nine NA subtypes recognized.^1^ Two new HA (H17 and H18) and NA subtypes (N10 and N11) subtypes have been proposed based on recent identification of these subtypes in fruit bats.^2^, ^3^ Although influenza A viruses are found in a range of species, avian species, in particular waterfowl, are the primary reservoir. Avian influenza A subtypes, specifically highly pathogenic avian influenza virus (HPAIV), have been the focus of many epidemiology studies as they are known to cause clinical disease in agricultural avian species. This ability to cause disease has at times led to severe ecological and in some cases economic impacts like what we are currently experiencing with the global spread of the HPAIV H5 clade 2.3.4.4b virus, which has caused deaths among wild and domestic bird species as well as a number of mammalian species.^4^ Swine are also thought to be central to the reassortment and cross‐species transmission of influenza A viruses, particularly to humans. The 2009 H1N1 pandemic virus is one such example of a swine origin virus that emerged and quickly spread through the human population in early 2009 and is now endemic.^5^

Considering the number of influenza A virus subtypes and numerous host species, there is a need for a diagnostic algorithm to efficiently and effectively screen and characterize samples from various host species and environments. To our knowledge, no such diagnostic algorithm exists in one place that broadly describes the best diagnostic workflow for screening samples based on host species. In this paper, we describe a diagnostic algorithm that can be used as a guide for screening human, animal, and environmental samples for influenza A viruses, specifically those viruses that are likely to have the highest health impact on human or animal populations.

## 2 METHODOLOGY AND RESULTS

**FIGURE 1:**
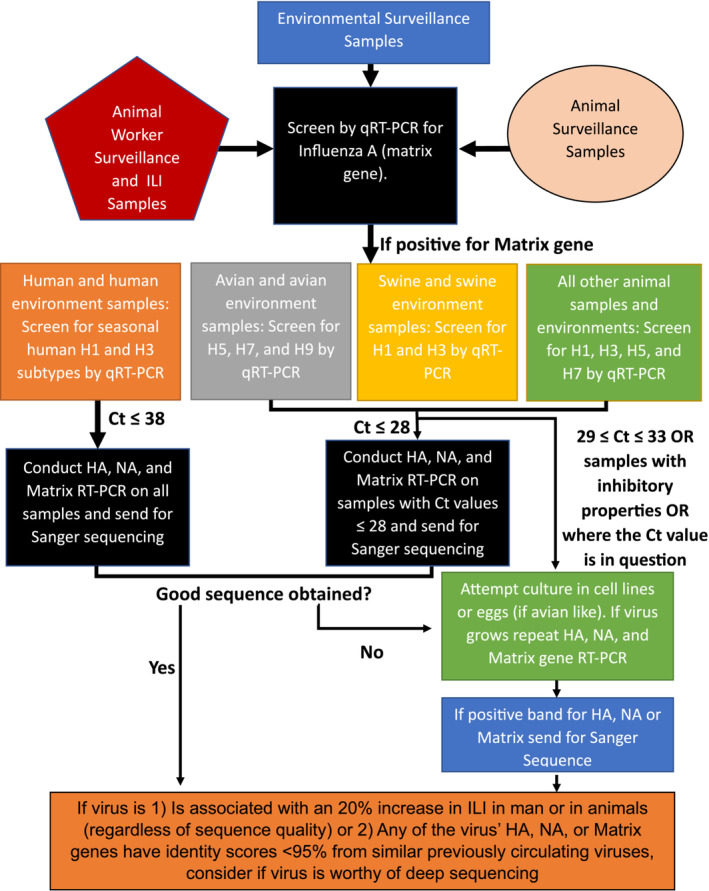
Detection algorithm for influenza A viruses in animal workers, animals, and the environment. Ct, Cycle threshold; HA, hemagglutinin gene; ILI, influenza‐like illness; NA, neuraminidase gene; qRT‐PCR, real‐time reverse‐transcriptase polymerase chain reaction; RT‐PCR, conventional reverse‐transcriptase polymerase chain reaction.

We searched the literature to determine influenza subtypes that were endemic in host species, such as H1 and H3 in human populations, as well as subtypes with increased disease severity in host species or propensity to cross‐species barriers, such as HPAIV H5 or H7 subtypes and categorized them broadly by animal type.^6^, ^7^, ^8^ Based on this information, we first considered a diagnostic algorithm that might be applied to humans, especially humans with animal exposures. Later we added nonhuman animal species as well as environmental samples to the algorithm (Figure 1). In particular, we sought to direct viral characterization to swine and avian‐reservoired viruses as influenza virus is highly enzootic in these species. We also incorporated environmental surveillance samples which could include bioaerosol samples, water samples, fecal samples, or environmental swabs, among others. Our general focus was to develop an algorithm that would be useful no matter the viral source (human, animal, or environment) and specifically to those viruses that have the greatest potential to cross‐species barriers or cause significant disease among host species.

In this algorithm, samples are first screened for the influenza A matrix gene, a general screening target for all influenza viruses, using the current CDC‐ or WHO‐approved primer and probe sets.^9^, ^10^ Samples that are positive for the matrix gene are next screened for various subtypes of interest based on their ability to cause significant disease (e.g. H5 or H7 subtypes, which are known to include HPAIV strains) or their epi‐ or enzootic nature in host species using approved qRT‐PCR protocols. For example, human and human environmental samples are first screened for human seasonal H1 and H3 influenza viruses. Avian and avian environmental samples are first screened for H5, H7, and H9 influenza viruses. Swine and swine environmental samples are first screened for H1 and H3 influenza viruses. All other animal samples are first screened for H1, H3, H5, and H7 strains. Some recommended primers and probes are available in the Supporting Information for these subtypes.

Based on these results, we then suggest that all influenza A matrix gene positive human and human environmental samples (Ct ≤ 38), regardless of positivity for seasonal human H1 and H3, be screened by conventional RT‐PCR for the HA, NA, and matrix genes and their amplicons, if of an expected molecular weight, be sent for Sanger sequencing for further characterization. Similarly, among animal and animal environmental samples, specimens with Ct values ≤ 28 should undergo conventional RT‐PCR for HA, NA, and matrix genes and be sent for Sanger sequencing for further characterization. If good sequence cannot be obtained from human, animal, or environmental samples by conventional RT‐PCR, culture should be attempted in either cell culture or eggs, if the subtype is suspected of avian origin. If virus grows, repeat conventional RT‐PCR for HA, NA, and matrix genes and submit for Sanger sequence. For samples with a high qRT‐PCR Ct value between 29 and 33, or for samples types that are known to have PCR inhibitory substances limiting PCR sensitivity, such as fecal samples,^11^ or for samples with high Ct values where the quality of RNA is in question, culture may be required in either cell culture or eggs first, where possible, to obtain good sequence data. In this instance, cultured samples should undergo conventional RT‐PCR for HA, NA, and matrix genes followed by Sanger sequencing. In our experience, Sanger sequencing studies of specimens with influenza A qRT‐PCR Ct values greater than 33 often fails. Hence, we suggest focusing characterization efforts on samples with Ct values ≤ 33. Based on sequence data obtained, samples should be considered as worthy of further study via deep sequencing if (1) the influenza virus identified is associated with a 20% increase in influenza‐like illness in humans or animals above historical baseline levels of disease (regardless of the sequence quality) or (2) if the Sanger sequence data for HA or NA genes have nucleotide identity scores <95% similarity with previously circulating subtypes in the host species.

## 3 DISCUSSION AND CONCLUSION

Apart from influenza A screening in human clinical samples, the authors do not know of any diagnostic algorithms that provide an overview of influenza A testing for both humans and other animal species. Hence, this study aimed to provide a broad influenza A diagnostic algorithm capturing samples from humans, animals, and their environments. Such an algorithm will allow laboratories to screen samples more effectively and efficiently for influenza A viruses of importance to both human and animal health. Based on reviews of the literature, we chose to focus our screening of influenza A viruses on subtypes of high importance to human or animal health such as those that caused endemic disease in host species or those with increased disease severity or propensity to cross species barriers.

Currently, researchers have to piece together diagnostic algorithms or rely on new technology such as next generation sequencing to characterize influenza A viruses. While next‐generation sequencing is becoming more cost effective, the use of such methods is still restricted to laboratories that have the equipment, funding, and technical capabilities to both run and analyze such data. This is particularly hampered in parts of the world where the equipment and technical capabilities are not currently available or are limited. Given our interest in subtypes of high importance to human or animal health such as H5 or H7, it is important from a veterinary and public health perspective to quickly identify such subtypes to effectively implement intervention strategies. Whole genome sequencing can take several weeks to run samples and process data, whereas characterization by RT‐PCR and Sanger sequencing can be done over a few days. For these reasons, we believe a diagnostic algorithm focused upon conventional/qRT‐PCR techniques is valuable to the research community.

This diagnostic algorithm is meant to serve as a guide for researchers studying influenza A viruses. Depending on the geographical location and host species for the samples, the specific RT‐PCR protocols used will vary. For example, researchers studying North American avian species may need to adjust the specific RT‐PCR protocols used in this algorithm, so they are specific to North American avian influenza virus lineages. Similarly, research focused in Asia will need to adjust the algorithm to include protocols specific to Asian avian influenza virus lineages. Government‐based influenza research protocols such as those provided for research use by the US Centers for Disease Control and Prevention^10^ or the World Health Organization^9^ can be used to determine the most suitable molecular protocols based on sample geographic location. We provide a list of example primers and probes that could be used for each subtype based upon host (Tables S1 and S2). Researchers may also adjust the detection algorithm accordingly to answer their research questions of interest. For example, it may not be necessary to conduct NGS sequencing on all H9‐positive samples from avian species if this subtype is highly enzootic in the population and the subtypes are consistent across the avian species and location from year to year in which case one may wish to only study a random number generated subset of H9‐positive samples. Hence, this diagnostic algorithm broadly serves as a guide for researchers doing influenza A virus research across multiple host species and can be adjusted accordingly based on geographical location and research questions of interest.

## AUTHOR CONTRIBUTIONS

**Laura Ann Pulscher:** Conceptualization; data curation; formal analysis; investigation; methodology; project administration; visualization; writing—original draft; writing—review and editing. **Richard John Webby:** Conceptualization; investigation; methodology; visualization; writing—review and editing. **Gregory Charles Gray:** Conceptualization; funding acquisition; methodology; resources; supervision; visualization; writing—review and editing.

## CONFLICT OF INTEREST STATEMENT

The authors have no conflicts of interest to declare.

### PEER REVIEW

The peer review history for this article is available at https://www.webofscience.com/api/gateway/wos/peer-review/10.1111/irv.13258.

## Supporting information


**Table S1:** Example Primers and Probes for the Detection of Human, Avian, and Swine Influenza Subtypes of Interest.
**Table S2:** Example Primers for the Characterization of Influenza A Virus (IAV) Hemagglutinin (HA), Neuraminidase (NA), and Matrix (M) Genes.

## Data Availability

Data sharing is not applicable to this article as no new data were created or analyzed in this study.
