# Supplementary material for: An algorithm for the characterization of influenza A viruses from various host species and environments
Source: Influenza Other Respir Viruses. 2024 Feb 22;18(2):e13258. doi: 10.1111/irv.13258 (PMC10883340; doi:10.1111/irv.13258)
Supplement: Supplementary file 1 — Table S1: Example Primers and Probes for the Detection of Human, Avian, and Swine Influenza Subtypes of Interest. Table S2: Example Primers for the Characterization of Influenza A Virus (IAV) Hemagglutinin (HA), Neuraminidase (NA), and Matrix (M) Genes. [file IRV-18-e13258-s001.docx]

**Supporting Information**

Pulscher LA, Webby RJ, Gray GC. An Algorithm for the Characterization of Influenza A Viruses from Various Host Species and Environments. Influenza and Other Resp Viruses.

**Table 1: Example Primers and Probes for the Detection of Human, Avian, and Swine Influenza Subtypes of Interest.**

| **Subtype** | **Sequence 5' - 3'** | **Reference** |
| --- | --- | --- |
| **Human Seasonal Subtypes** |  |  |
| **Seasonal H1N1 (pdm)** |  |  |
| *H1pdm-169-F* | AAACTATGCAAACTAAGAGGGGT | [1] |
| *H1pdm-297-R* | TGTTTCCACAATGTAGGACCA | [1] |
| *H1pdm-244-P* | Q670–CCAGAGTGTGAATCACTCTCCACA–BHQ2 | [1] |
| **Seasonal H3N2** |  |  |
| *H3h-1070Fw* | ATGGTTGGGAGGGAATG | [1] |
| *H3h-1167Rv* | TGCTGCTTGAGTGCTT | [1] |
| *H3h-1144dProbe* | FAM-CTGCTGCTTGTCCTCTTCCCT-BHQ1 | [1] |
| **Avian Subtypes H5, H7, H9** |  |  |
| **H5** |  |  |
| *H5-1012F* | TGGGTACCACCATAGCAATGAGCA | [1] |
| *H5-1155R* | AATTCCCTTCCAACGGCCTCAAAC | [1] |
| *H5-1042-P2* | CFO560–TGGGTACGCTGCAGACAAAGAATCCA–BHQ1 | [1] |
| *IAV-H5a-1658F* | GTT CCC TAG YAC TGG CAA TCA T | [2] |
| *IAV-H5a-1747R* | AAT TCT ARA TGC AAA TTC TGC AYT G | [2] |
| *IAV-H5a-1685FAM* | FAM-CTG GTC TAT CYT THT GGA TGT GYT CCA ATG-BHQ1 | [2] |
| **H7** |  |  |
| *CNIC-H7F** | AGAAATGAAATGGCTCCTGTCAA | [1] |
| *CNIC-H7R** | GGTTTTTTCTTGTATTTTTATATGACTTAG | [1] |
| *CNIC-H7P** | FAM-AGATAATGCTGCATTCCCGCAGATG-BHQ1 | [1] |
| *IAV-HA7-1617-F* | AAA TTG AGC AGY GGM TAC AAR GA | [2] |
| *IAV-HA7-1707-R* | AAA ACC ART CCC ATT RCA ATG GC | [2] |
| *IAV-HA7-1649.1-FAM* | FAM-TGG TTT AGC TTC GGG GCR TCA TGY TT_BHQ1 | [2] |
| **H9** |  |  |
| *H9-1538-F* | GGGTCAAGCTGGAATCTGA | [1] |
| *H9-1651-R* | TGGACATGGCCCAGAACAAGAA | [1] |
| *H9-1567p2* | Q670-TGTCGCCTCATCTCTTGTGVTTGCAA-BHQ2 | [1] |
| *IAV-H9-2F* | ATG GGG TTT GCT GCC | [2] |
| *IAV-H9-2R* | TTA TAT ACA AAT GTT GCA YCTG | [2] |
| *IAV-H9-2FAM* | FAM-TTC TGG GCC ATG TCC AAT GG-BHQ1 | [2] |
| **Swine Subtypes H1 and H3** |  |  |
| **H1** |  |  |
| *IAV-H1-115F* | ACA CAA TAT GTA TAG GYT AHC ATG C | [2] |
| *IAV-H1-199R* | GAG TGT GTY ACT GTY ACA TTC TT | [2] |
| *IAV-H1-147FAM* | FAM-TCD ACM GAC ACT GTW GAC ACA GTA CTN GA-BHQ1 | [2] |
| **H3** |  |  |
| *IAV-H3-1667-F* | TGG ATT TCC TTT GCC ATA TCA TG | [2] |
| *IAV-H3-1784-R* | ATR CAC TCA AAT GCA AAT GTT GCA | [2] |
| *IAV-H3-1753-FAM* | FAM-CTA ATG TTG CCT YTY TGG CAG GCC CAC AT-BHQ1 | [2] |
| *May only pick up Eurasian strains | |  |

**Table 2: Example Primers for the Characterization of Influenza A Virus (IAV) Hemagglutinin (HA), Neuraminidase (NA), and Matrix (M) Genes.**

| **Gene** | **Sequence 5' - 3'** | **Reference** |
| --- | --- | --- |
| **Universal Primers for HA Gene** |  |  |
| *Bm-HA-1* | TATTCGTCTCAGGGAGCAAAAGCAGGGG | [3] |
| *Bm-NS-890R* | ATATCGTCTCGTATTAGTAGAAACAAGGGTGTTTT | [3] |
| **Universal Primers for HA2 Fragment** |  |  |
| *HA-1144* | GGAATGATAGATGGNTGGTAYGG | [4] |
| *NS-890R* | AGTAGAAACAAGGGTGTTTT | [4] |
| **Universal Primers for NA 1, 2, 4, 5, and 8 Genes** |  |  |
| *Ba-NA-1* | TATTGGTCTCAGGGAGCAAAAGCAGGAGT | [3] |
| *Ba-NA-1413R* | ATATGGTCTCGTATTAGTAGAAACAAGGAGTTTTTT | [3] |
| **Universal Primers for NA 3 Genes** |  |  |
| *Bm-N3-1* | TATTCGTCTCAGGGAGCAAAAGCAGGTGC | [3] |
| *Bm-N3-1420R* | ATATCGTCTCGTATTAGTAGAAACAAGGTGCTTTTT | [3] |
| **Universal Primers for NA 6 Genes** |  |  |
| *Bm-N6-1* | TATTCGTCTCAGGGAGCAAAAGCAGGGTGAAAATG | [3] |
| *Bm-NS-890R* | See above Universal HA2 Fragment Primer | [3] |
| **Universal Primers for NA 7 Genes** |  |  |
| *Bm-N7-1* | TATTCGTCTCAGGGAGCAAAAGCAGGGTGATTGAGAATG | [3] |
| *Bm-NS-890R* | See above Universal HA2 Fragment Primer | [3] |
| **Universal Primers for NA 9 Genes** |  |  |
| *Bm-N9-1* | TATTCGTCTCAGGGAGCAAAAGCAGGGTC | [3] |
| *Bm-N9-1473R* | ATATCGTCTCGTATTAGTAGAAACAAGGGTCTT | [3] |
| **Universal Primers for Matrix Gene** |  |  |
| *Bm-M-1* | TATTCGTCTCAGGGAGCAAAAGCAGGTAG | [3] |
| *Bm-M-1027R* | ATATCGTCTCGTATTAGTAGAAACAAGGTAGTTTTT | [3] |
| **Universal Primers for All IAV Genes** |  |  |
| *MBTUni-12* | ACGCGTGATCAGCRAAAGCAGG | [5] |
| *MBTUni-13* | ACGCGTGATCAGTAGAAACAAGG | [5] |

**References**

1. WHO, *WHO information for the molecular detection of influenza viruses*, in [*https://cdn.who.int/media/docs/default-source/influenza/molecular-detention-of-influenza-viruses/protocols_influenza_virus_detection_feb_2021.pdf?sfvrsn=df7d268a_5#:~:text=GENERAL%20GUIDELINES-,Specimen%20selection,bronchial%20aspirate%20are%20also%20useful*](https://cdn.who.int/media/docs/default-source/influenza/molecular-detention-of-influenza-viruses/protocols_influenza_virus_detection_feb_2021.pdf?sfvrsn=df7d268a_5#:~:text=GENERAL%20GUIDELINES-,Specimen%20selection,bronchial%20aspirate%20are%20also%20useful)*.* 2021: WHO.

2. Hoffmann, B., et al., *Riems influenza a typing array (RITA): An RT-qPCR-based low density array for subtyping avian and mammalian influenza a viruses.* Sci Rep, 2016. **6**: p. 27211.

3. Hoffmann, E., et al., *Universal primer set for the full-length amplification of all influenza A viruses.* Arch Virol, 2001. **146**(12): p. 2275-89.

4. Obenauer, J.C., et al., *Large-scale sequence analysis of avian influenza isolates.* Science, 2006. **311**(5767): p. 1576-80.

5. Zhou, B., et al., *Single-Reaction Genomic Amplification Accelerates Sequencing and Vaccine Production for Classical and Swine Origin Human Influenza A Viruses.* Journal of Virology, 2009. **83**(19): p. 10309-10313.
